# Prenatal alcohol exposure before pregnancy awareness: a thematic analysis of online forum comments and misinformation

**DOI:** 10.3389/fpubh.2025.1525004

**Published:** 2025-02-19

**Authors:** Nessie Felicia Frennesson, Youssouf Merouani, Julie Barnett, Angela Attwood, Luisa Zuccolo, Cheryl McQuire

**Affiliations:** ^1^Tobacco and Alcohol Research Group, School of Psychological Science, University of Bristol, Bristol, United Kingdom; ^2^Department of Economic History, School of Economics and Management, Lund University, Lund, Sweden; ^3^Department of Psychology, University of Bath, Bath, United Kingdom; ^4^Health Data Science Centre, Human Technopole, Milan, Italy; ^5^Medical Research Council (MRC) Integrative Epidemiology Unit, Population Health Sciences, Bristol Medical School, University of Bristol, Bristol, United Kingdom; ^6^Centre for Public Health, Bristol Medical School, University of Bristol, Bristol, United Kingdom

**Keywords:** pregnancy, alcohol, pregnancy awareness, social media, misinformation, thematic analysis, prenatal alcohol exposure

## Abstract

**Background:**

Many women consume alcohol while pregnant before they are aware of the pregnancy, raising concerns about potential harms to the developing fetus. Official guidelines in the United Kingdom recommend abstinence throughout pregnancy, and many women turn to online forums for reassurance and information. However, online forums can also become a source of misinformation, potentially increasing confusion and anxiety among women who have consumed alcohol before pregnancy awareness. This study explored discussions about alcohol consumption before pregnancy awareness on Mumsnet to understand the nature of peer response and assess the accuracy of information against official health guidelines and the scientific literature.

**Methods:**

A thematic analysis was conducted on 71 thread starts and 1,281 comments from Mumsnet. Data was collected via web scraping, followed by manual screening. Themes were identified, and information-sharing posts were fact-checked against scientific evidence and guidelines.

**Results:**

Two overarching themes with five sub-themes emerged: “Type of reassurance offered”, where users offered reassurance to alleviate worries, and “Reactions to reassurance”, where some users appeared reassured while others did not. While many found reassurance, fact-checking revealed that the majority of the information was inaccurate, often underestimating the risks of prenatal alcohol exposure.

**Conclusions:**

Online forums may provide a source of peer support to those who have consumed alcohol before pregnancy awareness but frequently spread misinformation about alcohol use in early pregnancy. Health professionals should ensure pregnant women have access to accurate information alongside appropriate support to reduce anxiety and avoid the spread of harmful misinformation.

## Introduction

Estimates show that up to 60% of women consume alcohol between conception and the awareness of pregnancy (1). Global estimates suggest that more than 100,000 children are born with FAS (Fetal Alcohol Syndrome) and 630,000 with fetal alcohol spectrum disorder every year (2, 3). The diagnosis of FAS requires specific features, including growth retardation, characteristic facial features, central nervous system dysfunction, and neurobehavioral disabilities (4–6). FASD is a broader term that includes individuals with FAS as well as those affected by prenatal alcohol exposure who have neurobehavioural impairment without facial dysmorphology and growth deficiency (5). FASD is associated with negative long-term outcomes including problems with school, housing and employment contact with the criminal justice system, and problematic alcohol and illicit drug use (7).

Until 2016, the guidelines set by the National Institute for Health and Care Excellence (NICE) in the United Kingdom (UK) stated that pregnant women should avoid alcohol in the first 3 months of pregnancy, and if they choose to drink, they should not drink more than 1–2 units twice per week (8). The Chief Medical Officers (CMOs) updated these guidelines in 2016 to recommend abstinence from alcohol “if you are pregnant or think you could become pregnant” (9). These guidelines also state that if women have consumed small alcohol before pregnancy awareness, the risks of it affecting the baby are low and that they should contact their midwife if they have concerns (10). Some welcomed the updated guidelines (11) whilst others had concerns that the new guidelines could lead to feelings such as guilt and anxiety (12). These potential unintended consequences are important as prenatal stress can lead to adverse child outcomes, such as delays in motor and cognitive development (13) as well as obesity (14). Furthermore, maternal stress may increase the likelihood of prenatal alcohol use (15) and alcohol can be used as a coping strategy for stress experienced in pregnancy (16). Therefore, in addition to preventing harm from prenatal alcohol exposure, it is also important to help pregnant women manage their stress, including support to manage worries and anxiety about alcohol consumption during pregnancy (17).

Peer support has been shown to be important for women during and after pregnancy, showing a positive effect on their emotional wellbeing (18). The Internet is one of the mediums through which women can seek information and peer support during pregnancy. Studies have shown that most pregnant women use the Internet at least once a month to seek information about pregnancy, that they perceive this to be reliable, and rarely discuss this information with healthcare providers (19). Moreover, online support groups have been shown to be an effective way to manage worries during pregnancy (20) and to share experiences and information (21). The reasons to seek online peer support are many; some seek it due to limited access to offline support networks (22), whilst others seek it due to feelings of not being able to open up to professional support (18). However, there is still a need to examine how women respond to online peer support in relation to questions about alcohol consumption before pregnancy awareness.

There are also risks associated with reliance on online pregnancy-related information. Misinformation, defined as false or inaccurate information (23), is easily spread, and studies have shown misinformation related to pregnancy on multiple topics, including COVID-19 vaccines (24), nutrition (25), and breastfeeding (26). To our knowledge, until now, there has been no research on online misinformation about alcohol and pregnancy.

Mumsnet (https://www.mumsnet.com/), founded in 2000, is one the most prominent online forums for parents in the UK, with around 8 million users (27). Since many people use social media and the Internet to seek health information (28) and to find support during pregnancy (29), Mumsnet affords an excellent opportunity for researchers to investigate unmediated opinions and thoughts about alcohol consumption during pregnancy. Online forums and social media are especially good for analyzing informal conversations, as they allow researchers to study how people interact with each other outside of research and clinical settings (30) and to investigate the spread of misinformation (31).

This study aimed to investigate online interactions between people who report drinking alcohol before awareness of pregnancy and those who respond to their posts, with a particular focus on the nature and reported outcomes of peer response. It also aimed to examine the extent to which information given by parenting forum users is consistent with official guidelines and evidence from the scientific literature.

## Materials and methods

The study is reported following the Standards for Reporting Qualitative Research guideline (32).

### Data source

Mumsnet allows members to post anonymously on its online forum, “Mumsnet Talk,” which consists of various subforums, including the “Pregnancy” subforum used in this study. Mumsnet users can start threads, typically by asking a question, and other users can respond by adding comments. Each user is identified by a unique username. While the forum is publicly accessible for viewing, an account is required for users to post content.

### Eligibility criteria

Eligible data included original, non-duplicate thread-starts and comments retrieved from the “Pregnancy” subforum of Mumsnet that discussed alcohol use prior to pregnancy awareness. Posts from users indicating they were under 18 years old at the time were excluded from the analysis. Threads posted after the updated CMO guidelines (9), 1 January 2016, up until the date of data collection, 22 November 2023, were analyzed.

### Data collection and screening

We carried out web-scraping in November 2023, using Python to collect thread starts and related comments on alcohol within the “Pregnancy” subforum of Mumsnet. Specifically, thread-starts that mentioned “alcohol” in the title or the post were collected. NFF manually screened the content of collected threads and excluded those that were unrelated to alcohol consumption before pregnancy awareness. Comments marked as “Message withdrawn at poster's request” were filtered out. NFF extracted data on thread starts and subsequent comments, username, date and time of the posts, and the post content and saved these in a custom Excel file template. NFF removed any thread starts without replies during the data-cleaning process, as the focus on this study was on peer interactions.

### Analysis

For the first part of this study, we conducted a qualitative inductive thematic analysis of Mumsnet posts following the recommended steps provided by Clarke et al. (33). The ontological approach adopted in this study was rooted in a critical realist stance, recognizing both an objective reality as well as the influence of social and historical contexts on our understanding of it. NFF read all the threads and comments to become familiarized with the data. NFF generated code labels and shared these, alongside the original data, with the research team for input. Following this, NFF derived initial themes to capture patterns within the data. The themes were reviewed, refined and discussed continuously amongst the researchers AA, CM, JB, and LZ until they were deemed to provide a meaningful and accurate summary of the data.

For the second part of the study, all the statements posted by users that contained an element of information-sharing were gathered. A summary of all the statements was created and fact-checked. The statements were fact-checked against what the research team deemed to be the most robust sources of evidence, including recent systematic reviews, official policy and health guidelines, and publications in leading journals by established authors in the field, these sources can be found in Table 1. This helped to ensure accuracy and up-to-date assessment of the evidence. The statements were deemed to either be generally supportive of the statement made or generally not supportive of the statement made. A detailed table of all the statements and the number of times they were made between 1 January 2016–22 November 2023, was created and can be found in the Supplementary material.

**Table 1:** Summary and appraisal of health information statements.

**Statement**	**Evidence generally supportive of the statement**	**Evidence generally not supportive of the statement**	**Source**
**Patterns of PAE**			
Damage to the baby/FAS/FASD/is from being an alcoholic/heavy drinking/regular drinking.		X	There is no known safe limit of alcohol consumption during pregnancy (34, 35).
Alcohol is only damaging at later stages of pregnancy.		X	PAE can be damaging during all stages of pregnancy (36).
Damage from alcohol is really rare.		X	Estimates have shown that 1 in 67 women who consume alcohol during pregnancy gives birth to a child with FAS, and 1 in 13 give birth to a child with FASD (2, 3). Prenatal alcohol exposure poses an increased risk of low birth weight, preterm birth, and being small for gestational (35)
**Brain**			
The baby does not have a brain at week XX (comments ranging from weeks 4–6), so the alcohol does not damage the baby.		X	The neural tube that later becomes the baby's brain starts developing at week 5 (37), and research has shown that drinking in the periconceptual period and in the first trimester is associated with adverse birth outcomes (38).
**Blood supply**			
The baby does not share blood supply at week XX (comments ranging from weeks 2–14), so the alcohol does not damage the baby.		X	The blood supply starts at around week 4–5 of the pregnancy (37). However, alcohol can still reach the embryo via active transport and diffusion (39).
**Yolk sack**			
The baby is living off the yolk sack at week XX (comments ranging from weeks 4–10), so the alcohol does not damage the baby.		X	The baby has its own yolk sac for the first trimester (40). However, alcohol can still reach the embryo via active transport and diffusion (39).
**Placenta**			
The baby is not connected to the placenta at week XX (comments ranging from 2 to 14), so the alcohol does not damage the baby.		X	The placenta is functional at weeks 10–12 (39). However, alcohol can still reach the embryo before that via active transport and diffusion (39)
The placenta will filter out everything damaging, so the alcohol does not damage the baby.		X	The placenta can filter out some toxins (41). However, the placenta does not filter out alcohol (42).
**Implantation**			
Baby is not implanted at week XX (comments ranging from weeks 2–4), so the alcohol does not damage the baby.		X	The implantation happens around day 5–6 (37). Moreover, studies have shown that drinking in the periconceptual period can lead to spontaneous abortion (43).
**Heart**			
Baby does not have a heartbeat at week XX (comments ranging from weeks 2–5), so the alcohol does not damage the baby.		X	At week 6, a heartbeat can sometimes be detected during vaginal ultrasounds (37). However, studies have shown that both drinking in the periconceptual period and in the first trimester have associations with adverse birth outcomes (38).
**General**			
It is just a bunch of cells at week XX (comments ranging from 2 to 6), so the alcohol does not damage the baby.		X	Up until week 8, the developing baby is still considered to be an embryo and not a fetus (37). However, studies have shown that both in the periconceptual period and in the first trimester have associations with adverse birth outcomes (38).
Just don't drink in the second half of the first trimester.		X	Studies have shown that both drinking up to the pregnancy and in the first trimester have associations with adverse birth outcomes (38).
Other countries are not as strict with alcohol and pregnancy as the UK.		X	Most countries recommend abstinence during pregnancy (44).
Public health messaging is just cautious.	X		Based on the need for clarity and simplicity in providing helpful advice for pregnant people and the uncertainties that exist about any completely safe level, the CMO guideline recommended a “precautionary” approach that it is safest to avoid drinking any alcohol in pregnancy (35)
There is no actual research on alcohol and pregnancy.		X	There have been several large reviews showing the prevalence and effects of alcohol in pregnancy (2, 4). Research using animal studies has also shown that moderate prenatal alcohol exposure can lead to neurobehavioral effects in offspring (45). A search of the terms alcohol AND pregnancy on the PubMed database for research literature in October 2024 revealed 32,909 results.^a^

PAE, prenatal alcohol exposure; FAS, Fetal Alcohol Syndrome; FASD, fetal alcohol spectrum disorder.

a https://pubmed.ncbi.nlm.nih.gov/?term=%28alcohol%29+AND+%28pregnancy%29.

### Ethics

Ethics approval was granted by the School of Psychological Science Research Ethics Committee at the University of Bristol in August 2023 (Ethics approval code 14455). To safeguard anonymity, we removed personally identifiable information during data cleaning. In adherence to guidelines outlined by the British Psychological Society (46) we do not include direct quotations. All the quotes presented in this study were paraphrased.

## Results

After applying the eligibility criteria, we included 71 thread starts and 1,281 related comments in our analyses.^1^ All comments belonged to thread starts in which forum users reported consuming alcohol before pregnancy awareness and expressed some kind of worry or anxiety about this. In general, all thread starts included information on how much the thread starter had drunk before pregnancy awareness as well as what week of the pregnancy they believed they were in. A minority of thread starts reported heavy drinking whilst most thread starts reported one or few situations where they had been drinking a small amount of alcohol or had been drinking on one or two occasions. A more detailed description of the thread-starts is presented by Frennesson et al. (47).

### Thematic analysis

Our analysis resulted in two overarching themes: (1) “Type of reassurance offered,” (2) “Reactions to reassurance.” These themes had the following sub-themes: (1) “Emotion-based reassurance,” “Information-based reassurance,” “Lived experience-based reassurance,” and “Reassuring but with concerns,” (2) “Reassured” and “Unconvinced by reassurance.”

#### Type of reassurance offered

##### Emotion-based reassurance: “No one is perfect”

This sub-theme captured the emotional-based reassurance provided by those who commented on the thread starts. Many of those who commented offered emotional responses to alleviate the anxiety the thread starters felt about having consumed alcohol before pregnancy awareness. This response often involved normalizing the anxiety the thread starter felt and also encouraging the thread starters to focus on the positive aspects of the pregnancy: “*What is done is done. No one is perfect, so you will just have to do your best to manage your worries so just try to enjoy your pregnancy! It is such an amazing time in your life so do not waste it on having anxiety about something that you cannot change.”*

Practical advice was also commonly shared, with suggestions to seek early scans or consult healthcare providers: “*Try to get an appointment with your midwife; they might suggest you have an early scan. Honestly, that helped me so much with my worries as they are there to help you.”* Advice such as this provided the thread starters with actionable steps to alleviate their concerns at the same time as promoting open communication with healthcare professionals.

General reassurance and positive affirmations were also prevalent, with comments such as: “*You so deserve this baby. Don't hate yourself, please and good luck!”*. These messages offered support and empathy, potentially helping thread starters feel less isolated in their concerns.

Lastly, some comments appeared to downplay the potential harm of occasional alcohol consumption, with reassurances like, “*Honestly, you had a few drinks on a few nights out, it is not like you used heroin is it?”*. These comments seemed to try to provide a more balanced perspective, aiming to reduce the anxiety felt by the thread starters.

Overall, this sub-theme highlighted the reassurance provided by online interactions offering empathy as well as practical advice for those concerned about alcohol consumption before pregnancy awareness.

##### Information-based reassurance: “The baby does not even have a brain yet”

This sub-theme captured responses that shared medical advice regarding alcohol consumption in early pregnancy. These frequently related timing of exposure to developmental stages to explain why alcohol would not be harmful to ease the anxiety of the thread starter. For example, comments often mentioned that the baby is not connected to the placenta or bloodstream until later in the pregnancy: “*You're fine; the baby isn't even connected to the placenta until week 7, so honestly, nothing to worry about. And even after that, the placenta is there to filter out all the bad stuff before it reaches the baby”* These comments usually provided specific, but almost never factually correct, developmental information but did not provide where this information came from. Table 1 provides further analysis of the accuracy of such information.

There was also skepticism about existing research on alcohol in pregnancy: “*You know what, they don't actually know anything because it is not like they can do experiments on babies and alcohol can they? They are just being overly cautious because it is easier for them.”* These comments highlighted some distrust in the current research and guidelines, including concerns about validity due to ethical limitations in studying pregnant women and the dismissal of recommendations that adhere to the precautionary principle.

Additionally, many shared that their midwives had reassured them that drinking early in pregnancy is usually not a concern: “*My midwife almost laughed at me when I said that I was worried about drinking before I knew I was pregnant, she said that A LOT of babies are conceived on a heavy night out so nothing to worry about. Just relax and enjoy!”* Comments like these suggested some variability in healthcare providers' communication and support strategies around periconceptual alcohol exposure.

Overall, this sub-theme highlighted the role of medical advice, usually based on anecdotal exchanges with healthcare professionals or forum users' own interpretation of information on prenatal development, with the apparent intention of reducing the thread starter's anxiety. In the next section, we explore the nature and validity of the information shared in this sub-theme.

##### Lived-experience based reassurance: “My baby turned out fine”

This sub-theme covered those comments who shared their own experience of consuming alcohol before pregnancy awareness. These comments frequently highlighted that their children were fine: “*It is nothing to worry about. I went on a massive binge (honeymoon) before I knew that I was pregnant and my baby boy is now doing his A-levels and is absolutely acing it.”* Anecdotes like these sought to provide reassurance by showing positive outcomes despite their early alcohol consumption.

Other responses mentioned that older generations had a different approach to drinking during pregnancy and that it was seen as nothing bad and more accepted: “*Listen, my grandmother and even my mum used to drink throughout the pregnancy and I am guessing that so did yours. We all turned out fine didn't we?”* These comments reflected a more cultural/intergenerational perspective, highlighting other's experiences, implying that drinking before pregnancy awareness might not be a cause for concern or anxiety.

Overall, this theme highlighted how those commenting often used personal experiences and highlighting positive outcomes, to reduce worries and provide reassurance to the thread starter.

##### Reassuring but with concerns: “It is not ideal”

Although most comments offered reassurance, some also expressed concerns about the thread starters' situations, mainly when binge or heavy drinking was involved during a prolonged period. The comments in this theme varied, with some highlighting the uncertainty of the baby's health until birth and others advising seeking medical help for anxiety: “*Try to relax because worrying won't get you anywhere. There is actually no way of knowing if you have harmed your baby until it is born, so just do what you can to prepare yourself for a possible reality where it might have some damage”*. Comments like these reflected that there are potential risks with drinking before pregnancy awareness, particularly for higher levels of alcohol exposure, indicating a need for preparation and acceptance of possible outcomes.

Others acknowledged that drinking during pregnancy is potentially harmful: “*You should talk to your midwife, drinking EVERY day is not great, but I am happy to hear that you have stopped not, it could not have been easy”*. These comments reflected how consuming alcohol could have some potential risks while recognizing that abstinence can be challenging for some.

Overall, this theme highlighted responses which expressed concerns at the same time as offering some reassurance and understanding. This concern was usually targeted toward those who admitted to binge drinking or being heavy drinkers, not including those who had an occasional drink or had been drinking due to celebrations such as Christmas or honeymoons.

#### Reactions to reassurance

##### Reassured

This sub-theme captured both the thread starters who later returned to the thread expressing reassurance and those commenting on the thread, finding reassurance in knowing that others had been in similar situations. Comments from the former often described feelings of relief and support: “*I am so relieved to hear that I am not the only one who has done this and that your babies all turned out fine. I wish I had turned to you guys before going into panic mode!”*. Comments like these suggest that knowing others had similar experiences and positive outcomes helped to alleviate some anxiety.

Comments from the latter, often highlighted how reading about other's experiences reduced their anxiety about consuming alcohol before pregnancy awareness: “*Thank god for this thread, it makes me feel so much better to hear that all of your babies turned out fine. I have also been drinking for the first few weeks and was panicking before I read this thread!”*. Comments like these illustrate how shared experiences and worries may create a sense of mutual support and reassurance.

Moreover, some asked the thread starter for updates, looking for longer-term reassurance about their situations: “*I just found out that I am pregnant and I am having the same worry as you, I know that this thread is a few years old so could you tell us if your baby turned out fine or not?”*. Comments like these suggest a need for reassurance from others who have been through a similar situation and an investment in finding out about longer-term outcomes.

Overall, this theme highlights the importance of peer response in providing reassurance to those concerned about alcohol consumption before pregnancy awareness.

##### Unconvinced by reassurance

This theme captured how some of the thread starters continued to have worries. These worries persisted even after hearing reassurance from other forum members: “*Thank you all for your replies. I feel like the worry is slowly eating me up, but having a meeting with my midwife tomorrow so hopefully that will help”*. Comments like these indicate that, despite peer response, the worry remained, highlighting the potential need for professional support.

For some of the thread starters, the worry was not eased after seeing health professionals: “*I just had my scan, and everything looked fine, but I'm still so worried about fetal alcohol spectrum disorder (FASD)!”*, This reflecting that even after both peer reassurance and reassurance from healthcare providers, worry and anxiety can still be present.

Overall, this theme shows that the anxiety was not always relieved among thread starters, despite the peer reassurances provided. This highlights the need for professional guidance and support.

### Health information shared in theme “Information-based reassurance”

Table 1 provides a summary and appraisal of the statements posted in the sub-theme “Information-based reassurance”. It outlines whether evidence or official guidance from the field generally supports or does not support the statements made. The source column shows the sources that we used to assess the statements. A detailed version of this table with all the comments, as well as the number of comments, can be found in the Supplementary material. Out of 1,281 comments, 16.08% (206) of those contained information-based reassurance.

## Discussion

This study provided novel insights into the nature of online peer exchanges about prenatal alcohol exposure that has occurred prior to pregnancy awareness. The seeking and offering of reassurance was the key overarching theme across exchange on the MumsNet forum. As well as emotional support, and anecdotal evidence forum users often offered information about the risks, or indeed perceived “safety” of alcohol exposure, however this information was often inaccurate and in contradiction to official guidance, or the current evidence base.

The analysis resulted in two overarching themes: (12) “Type of reassurance offered”, and (34) “Reactions to reassurance”. These themes had the following sub-themes: (12): “Emotion-based reassurance,” “Information-based reassurance,” “Lived experience-based reassurance,” and “Reassuring but with concerns”, (34): “Reassured” and “Unconvinced by reassurance”.

The themes expressed sympathy for the thread starters, often relying on personal experiences without providing substantial evidence. Although reassurance was predominant, some comments also expressed concerns about alcohol consumption, particularly when the thread starter had reported binge or heavy drinking. These comments often emphasized the potential risks, but were largely supportive and empathetic in nature, suggesting that the thread starter should seek further professional advice. Forum users' reactions to reassurance often indicated gratitude, with users often reporting that they felt reassured by others' experiences and advice. However, some thread starters continued to experience anxiety and worry despite the peer response received and sometimes despite support from healthcare professionals, highlighting the need for improved guidance and support.

Given the growing concern about misinformation related to pregnancy in online forums (48), the study also examined the accuracy of the information shared by forum users about the risks of drinking alcohol before pregnancy awareness. Inaccuracies were common, including common misconceptions about the risks of prenatal alcohol exposure at different stages of pregnancy and at different levels of exposure.

Mumsnet has previously been shown to help investigate topics around pregnancy and motherhood. It has, for example, been used to explore experiences of breastfeeding (49) and maternal feelings (50). In line with previous research, this study demonstrated how women turn to online forums to look for support and knowledge (49), as well highlighting this as a process that can help reduce anxiety and give personal support (51). However, even though women found reassurance from others, this study demonstrated that the information shared is often incorrect. Previous studies have shown that misinformation is easily spread on the internet for topics such as COVID-19 vaccines in pregnancy (24), nutrition in pregnancy (25), and breastfeeding (26, 52).

This study evidently shows that people turning to Mumsnet for advice and reassurance are finding this. However, as this study has presented, the information shared related to alcohol consumption before pregnancy awareness was, in most cases, incorrect. This highlights the need for pregnant women to easily find the correct information online and have a place they can turn to with their questions and worries.

## Strengths and limitations

This study retrieved all available data related to drinking before pregnancy awareness dating from the updated CMO guidelines in 2016 up until the end of 2023 from the largest dedicated online parenting forum in the United Kingdom. Moreover, to our knowledge, this is the first study to provide insight into how people respond to those seeking information and reassurance after drinking before pregnancy awareness, how interactions on online forums may work to support pregnant women, as well as being the first study to examine what extent the information shared is correct. This study highlights the need for both policymakers and those providing health care to pregnant women to find a balance between providing correct information about alcohol at the start of the pregnancy at the same time as helping women manage their worries.

An important limitation is that the women posting on Mumsnet might not be representative of women who have consumed alcohol before pregnancy awareness. The demography of Mumsnet has previously been described as middle class and of higher education (53), meaning that voices from other social backgrounds are likely to be underrepresented. This is important since alcohol consumption during pregnancy has been shown to be related to higher education (36), and social media use is more common among those in higher socioeconomic groups (54). Patterns of prenatal alcohol use and the nature of social media interactions are likely to differ across population subgroups.

## Conclusion

This study provides valuable insights into the peer response and reassurance pregnant women can gain from online forums, revealing that peer reassurance often draws upon anecdotes and personal experiences rather than accurate health information. A common misconception was about the risk of PAE at the different stages of the pregnancy. These findings show the importance of ensuring that pregnant women have access to accurate health information related to alcohol consumption before pregnancy awareness, alongside emotional support and reassurance. This study also highlights the need for policymakers and healthcare professionals to balance providing accurate information while helping pregnant women manage their worries.

## Data Availability

The datasets presented in this article are not readily available because the site contents are copyright of Mumsnet, publishing a data set collecting posts or threads is not possible. Instead, the summary statistics or analysis results will be published via data.bris. Requests to access the datasets should be directed to felicia.frennesson@bristol.ac.uk.

## Footnotes

^1^One thread start and six accompanying comments were excluded because the thread starter reporting having experienced a miscarriage. One further thread start and six accompanying comments were excluded due to replies only concerning drug use.
