# Supplementary material for: Prenatal alcohol exposure before pregnancy awareness: a thematic analysis of online forum comments and misinformation
Source: Front Public Health. 2025 Feb 19;13:1525004. doi: 10.3389/fpubh.2025.1525004 (PMC11883443; doi:10.3389/fpubh.2025.1525004)
Supplement: Supplementary file 1 [file Table_1.docx]

|  |  |
| --- | --- |
| **Statement** | **Number of statements (N=206)** |
| ***Patterns of PAE^1^*** |  |
| Damage to the baby is from heavy drinking. | 26 |
| FAS^2^/FASD^3^ is from being an alcoholic or heavy drinking. | 18 |
| FAS/FASD is from regular drinking. | 11 |
| Damage from alcohol is really rare. | 5 |
| Alcohol is only damaging at later stages of pregnancy. | 3 |
| Scans do not pick up on FAS. | 3 |
| You will usually know if your baby has FAS; they have distinct facial features. | 3 |
| 60% of alcoholics have babies that turn out fine. | 1 |
| The number of drinks causing FAS is not known. | 1 |
| Only 1-2% of pregnancies are affected by alcohol. | 1 |
| ***Brain*** |  |
| The baby does not have a brain at week 4+5. | 1 |
| The baby does not have a brain at week 6. | 1 |
| ***Blood Supply*** |  |
| The baby does not share blood supply for the first few weeks. | 6 |
| The baby does not share blood supply at week 2. | 2 |
| The baby does not share blood supply at week 3. | 4 |
| The baby does not share blood supply at week 4. | 1 |
| The baby does not share blood supply at week 5. | 2 |
| The baby does not share blood supply at week 4+5. | 1 |
| The baby does not share blood supply until week 5. | 1 |
| The baby does not share blood supply until week 6. | 4 |
| The baby does not share blood supply until week 8. | 1 |
| The baby does not share blood supply until week 10. | 1 |
| The baby does not share blood supply until weeks 10-14. | 1 |
| ***Yolk Sack*** |  |
| The baby is just living off the yolk sack at week 4. | 1 |
| The baby is just living off the yolk sack at week 7. | 1 |
| The baby is just living off the yolk sack for the first 10 weeks. | 1 |
| The baby has its own yolk sack during the first trimester. | 1 |
| The baby is living of the yolk sack until week 6. | 1 |
| ***Placenta*** |  |
| The baby is not connected to the placenta at weeks 2-3. | 1 |
| The baby is not connected to the placenta at week 4. | 1 |
| There is no placenta at week 4. | 4 |
| There is no placenta at week 5. | 5 |
| There is no placenta until week 6. | 2 |
| There is no placenta at week 6-7. | 1 |
| The placenta takes over at week 7. | 1 |
| The placenta takes over at week 7-8. | 2 |
| The placenta takes over at week 8-9. | 2 |
| The placenta takes over at week 9-10. | 2 |
| The placenta takes over at week 10-12. | 1 |
| The placenta takes over at week 12. | 2 |
| The placenta takes over at week 14. | 1 |
| There is no placenta until weeks 7-8. | 1 |
| There is no placenta early on in the pregnancy. | 1 |
| The placenta is not attached during the first couple of weeks. | 5 |
| The placenta will filter out all the bad stuff. | 1 |
| The placenta does not give nourishment until the end of the first trimester. | 1 |
| ***Implantation*** |  |
| The baby is not implanted at week 2. | 2 |
| The baby is not implanted until week 4. | 1 |
| ***Heart*** |  |
| The baby does not have a heartbeat at weeks 2-3. | 1 |
| The baby does not have a heartbeat at week 5. | 1 |
| ***General*** |  |
| Midwives or other health care providers said that alcohol consumption before pregnancy awareness is common or nothing to worry about. | 24 |
| There is no actual research on alcohol and pregnancy. | 7 |
| Other countries are not as strict with alcohol and pregnancy as the United Kingdom. | 4 |
| Public health messaging is just cautious. | 4 |
| It is just a bunch of cells at week 4. | 2 |
| It is just a bunch of cells at weeks 5-6. | 2 |
| Alcohol does not harm your baby at week 2. | 2 |
| You are barely pregnant at week 4. | 2 |
| The egg is just implanted at 4 weeks of pregnancy, so the baby will not be affected by alcohol. | 2 |
| Drinking before week 12 will cause miscarriage. | 2 |
| If it is early in pregnancy, you don’t share nutrients with the baby. | 2 |
| Occasional drinking does not harm your baby. | 2 |
| It is just a bunch of cells at week 2. | 1 |
| Alcohol does not harm your baby at week 4. | 1 |
| Alcohol cannot pass to the baby at week 4. | 1 |
| The umbilical cord is not attached until week 5. | 1 |
| Drinking 3-7 drinks a week will not increase the risk of learning difficulties. | 1 |
| The important time to not consume is week 6-12. | 1 |
| If it is early in pregnancy, you don’t transmit anything to the baby. | 1 |
| Just don’t drink in the second half of the first trimester. | 1 |
| 14 Units of alcohol per week during pregnancy is fine. | 1 |
| ^1^ Prenatal Alcohol Exposure  ^2^ Fetal Alcohol Syndrome  ^3^ Fetal Alcohol Spectrum Disorder |  |
|  |  |
